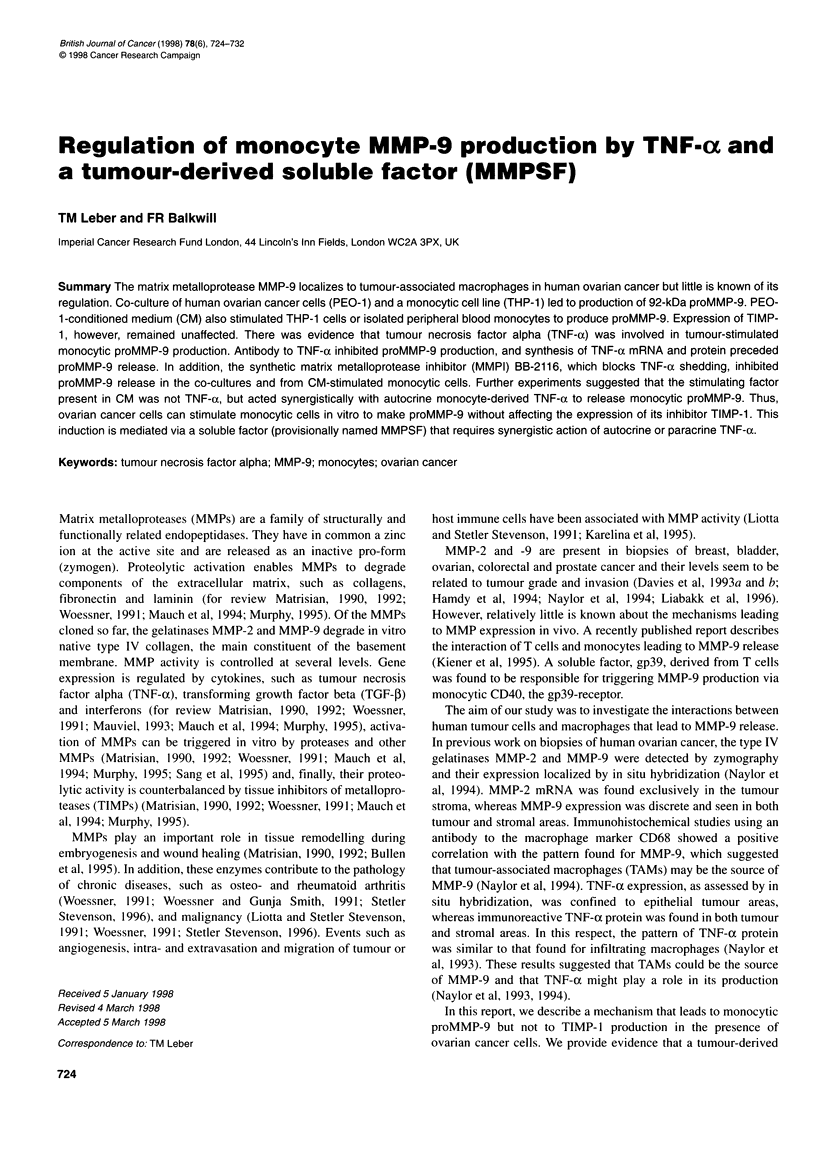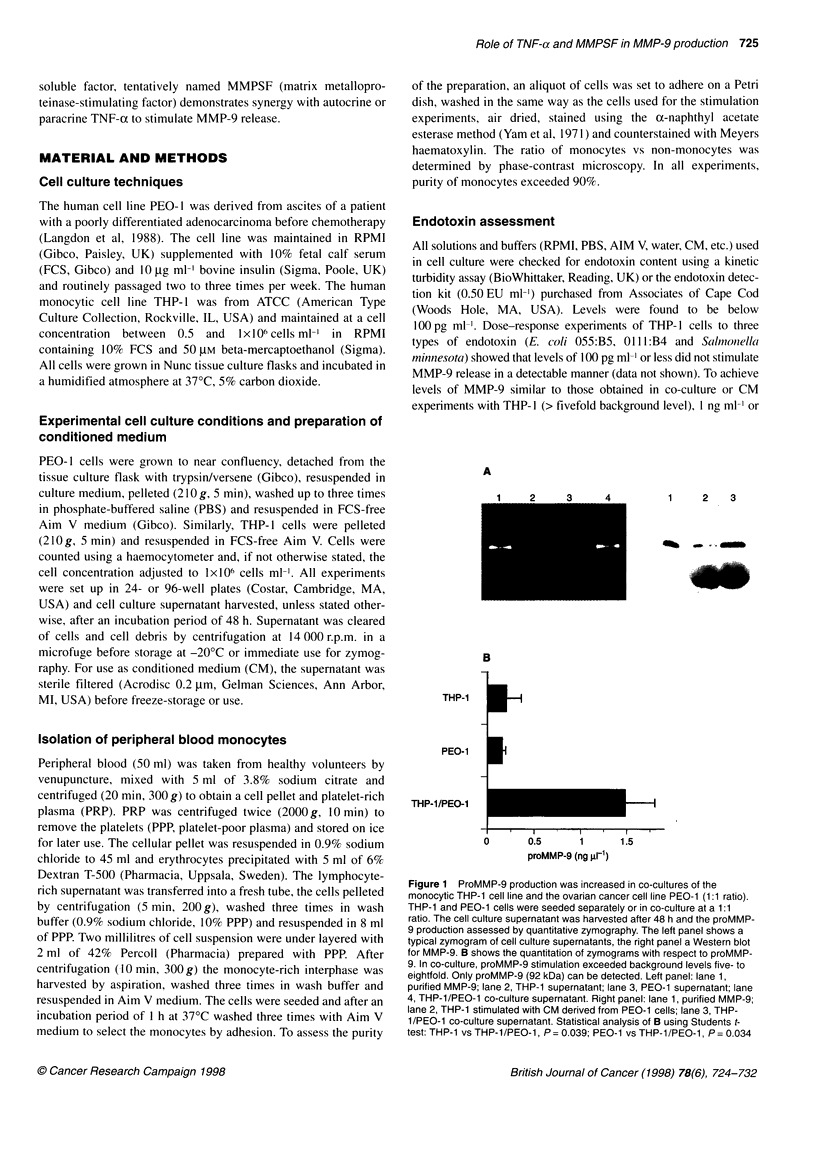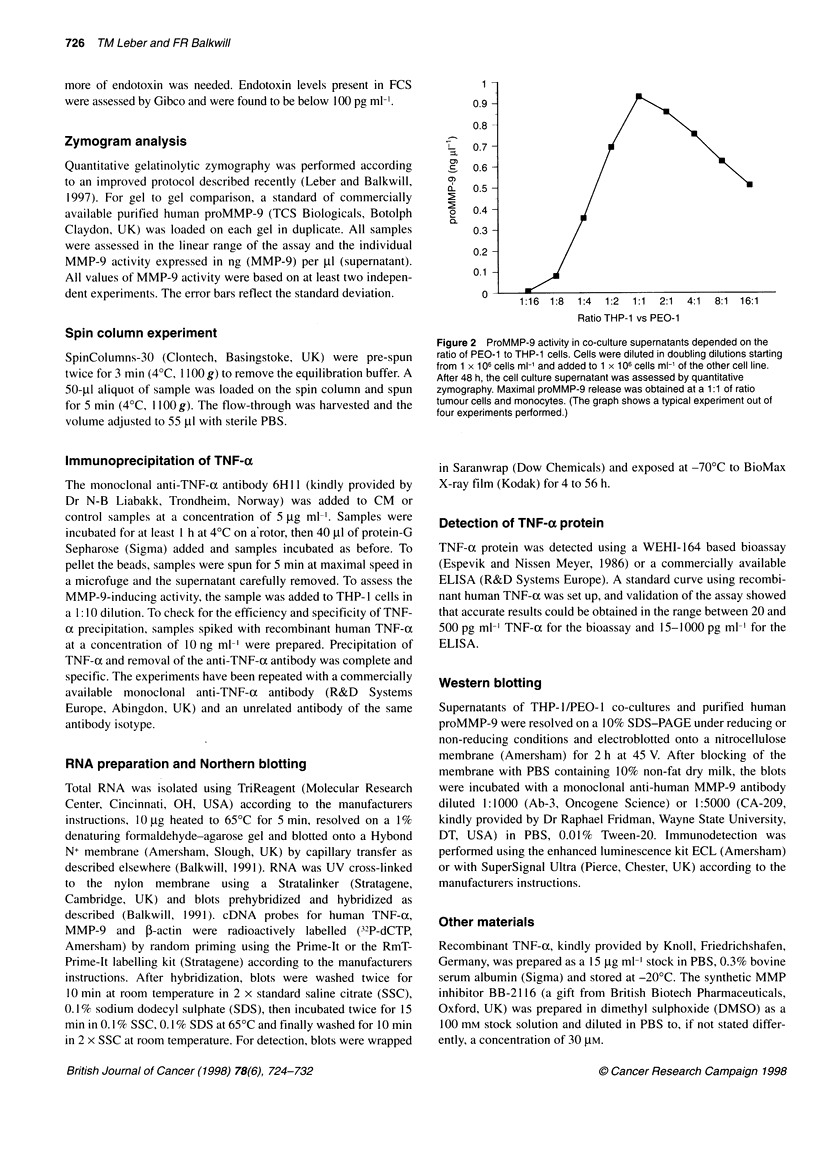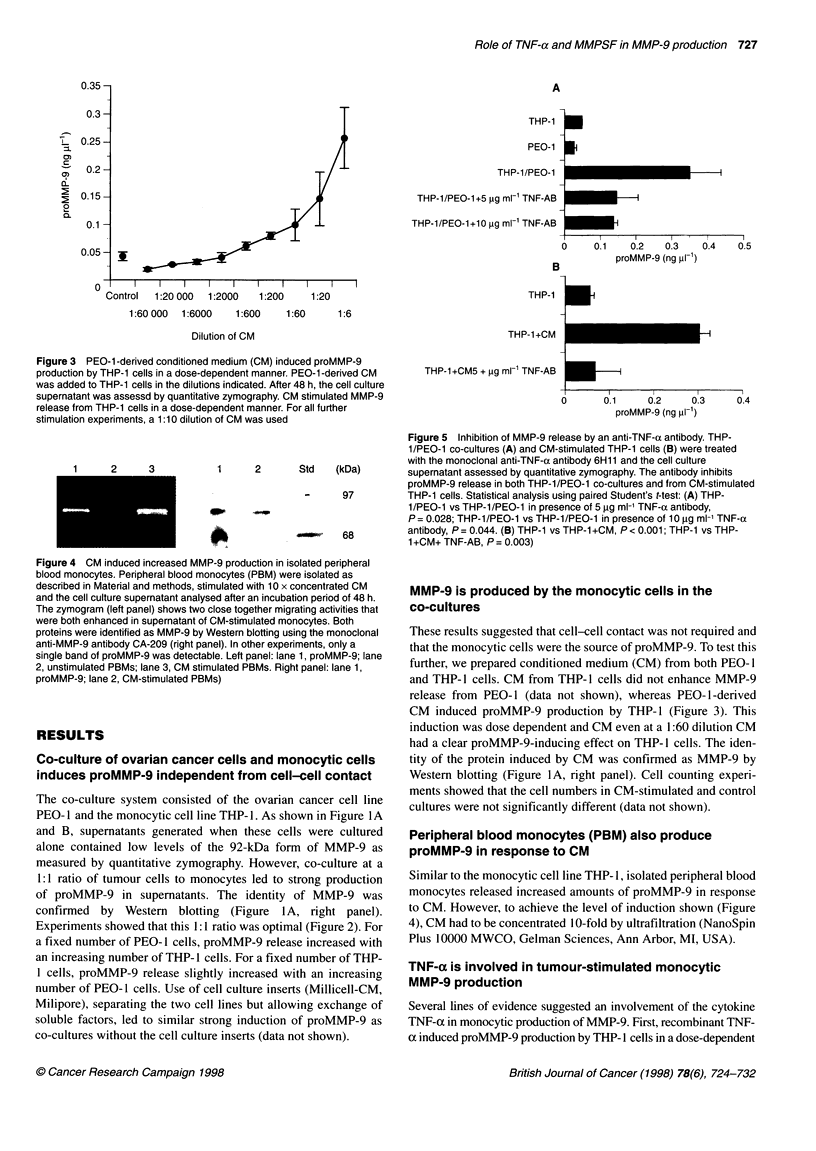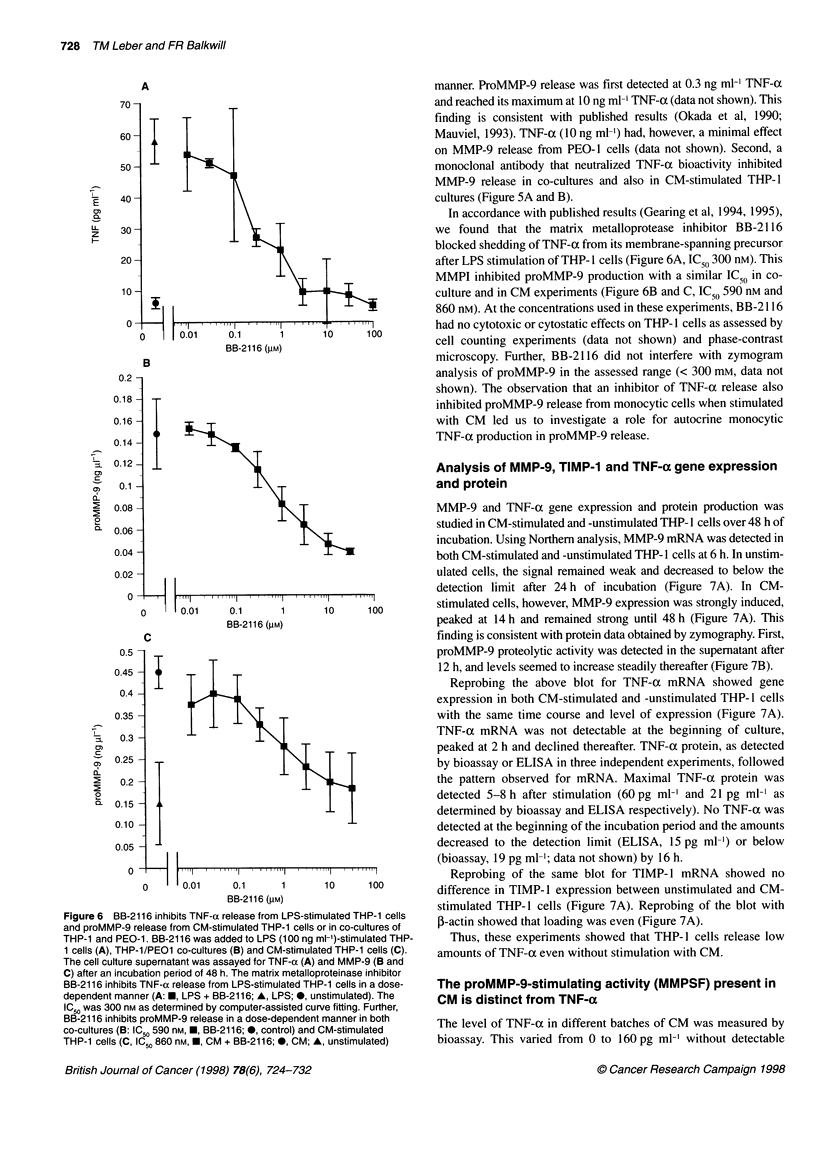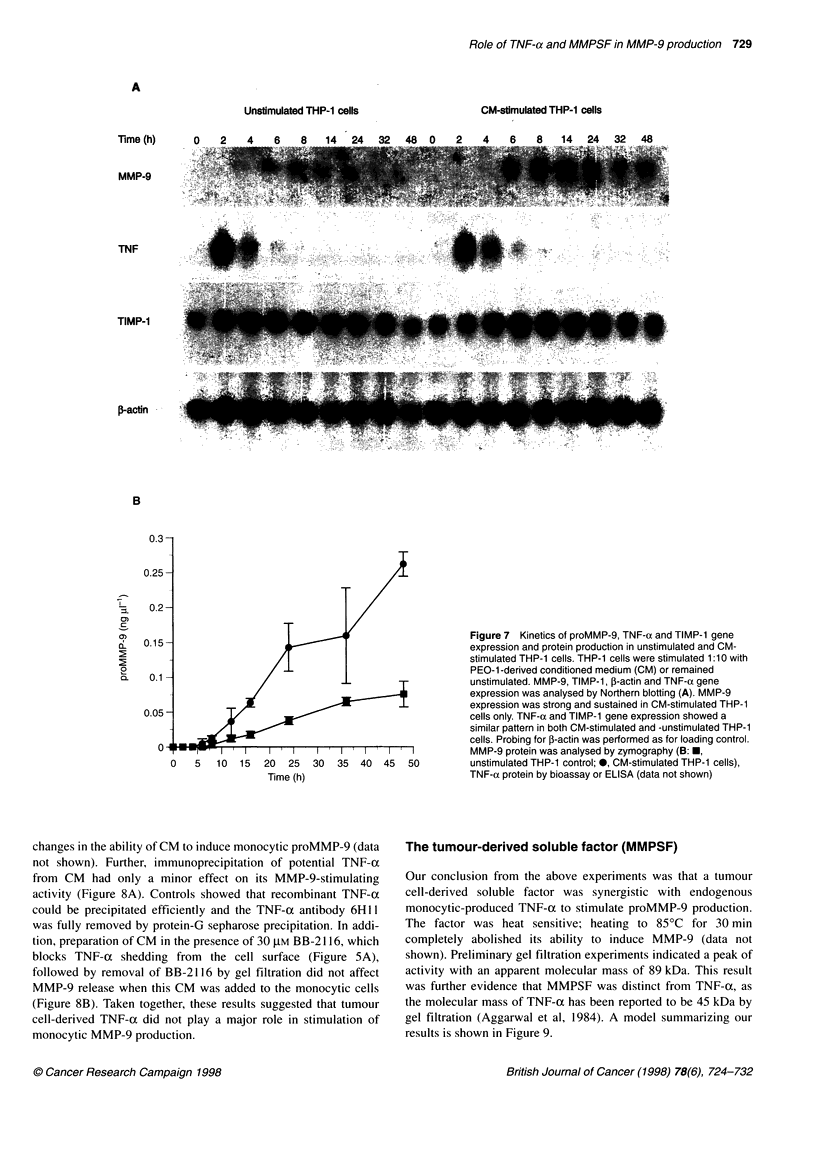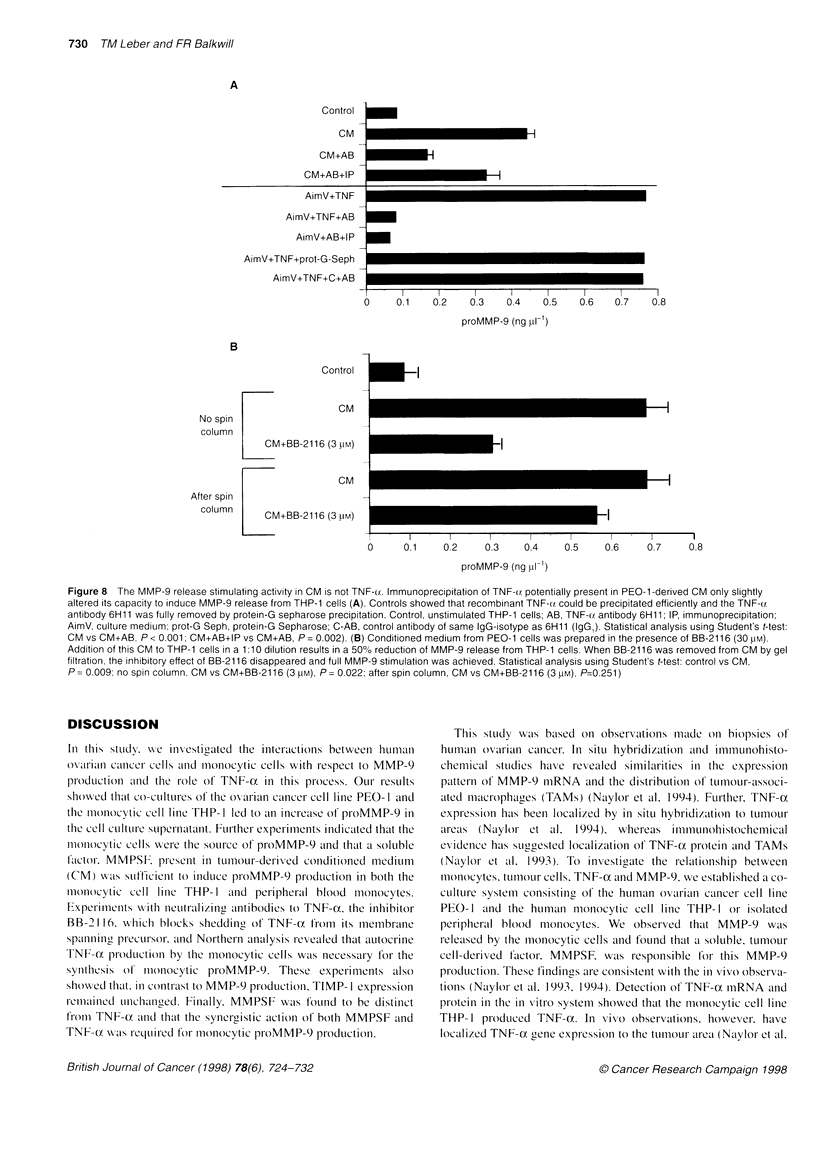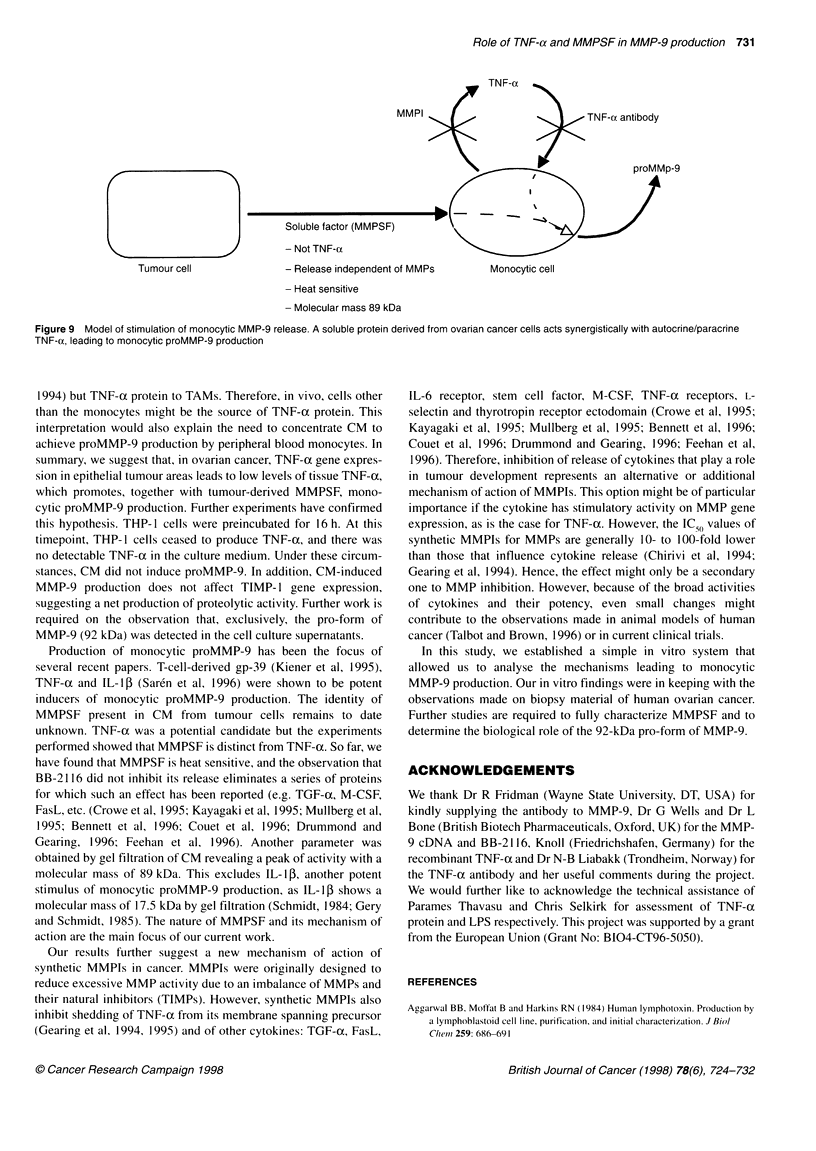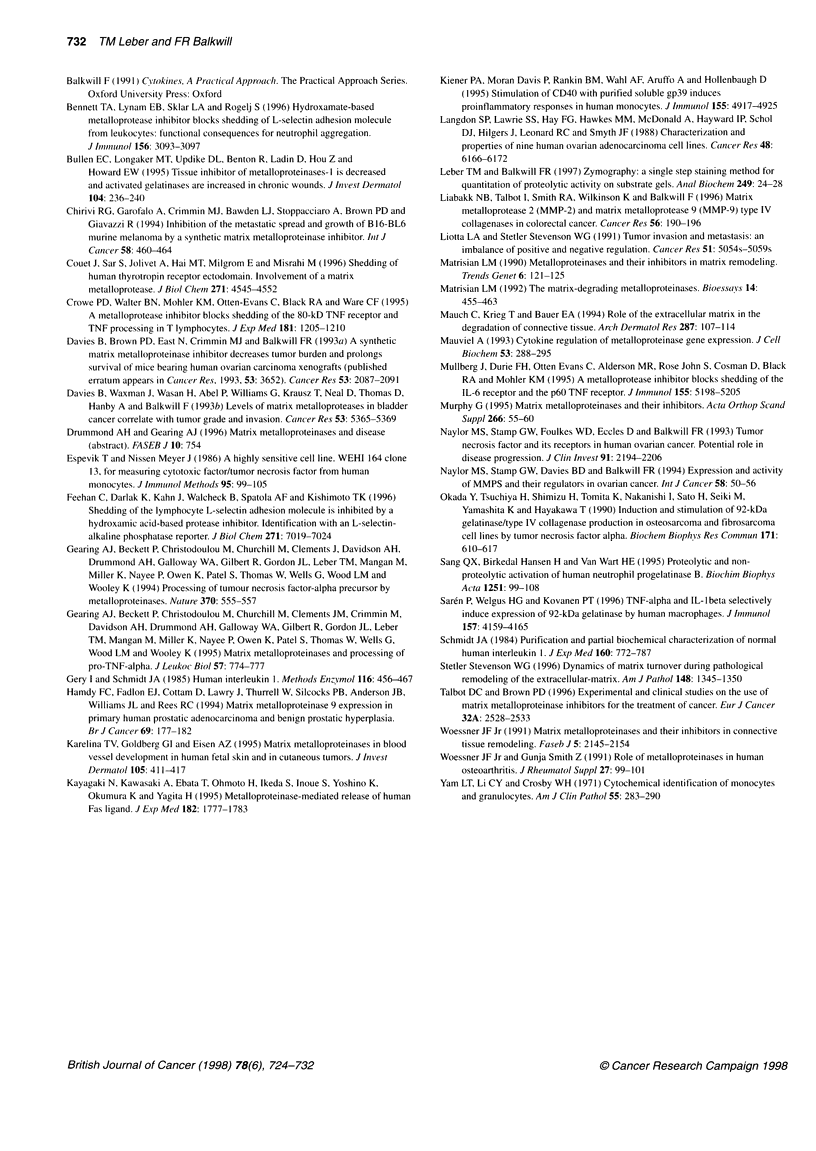# Regulation of monocyte MMP-9 production by TNF-alpha and a tumour-derived soluble factor (MMPSF).

**DOI:** 10.1038/bjc.1998.568

**Published:** 1998-09

**Authors:** T. M. Leber, F. R. Balkwill

**Affiliations:** Imperial Cancer Research Fund London, UK.

## Abstract

**Images:**


British Journal of Cancer (1998) 78(6), 724–732
© 1998 Cancer Research Campaign

### Regulation of monocyte MMP-9 production by TNF-α and a tumour-derived soluble factor (MMPSF)

**TM Leber and FR Balkwill**

Imperial Cancer Research Fund London, 44 Lincoln's Inn Fields, London WC2A 3PX, UK

**Summary** The matrix metalloprotease MMP-9 localizes to tumour-associated macrophages in human ovarian cancer but little is known of its regulation. Co-culture of human ovarian cancer cells (PEO-1) and a monocytic cell line (THP-1) led to production of 92-kDa proMMP-9. PEO-1-conditioned medium (CM) also stimulated THP-1 cells or isolated peripheral blood monocytes to produce proMMP-9. Expression of TIMP-1, however, remained unaffected. There was evidence that tumour necrosis factor alpha (TNF-α) was involved in tumour-stimulated monocytic proMMP-9 production. Antibody to TNF-α inhibited proMMP-9 production, and synthesis of TNF-α mRNA and protein preceded proMMP-9 release. In addition, the synthetic matrix metalloprotease inhibitor (MMPI) BB-2116, which blocks TNF-α shedding, inhibited proMMP-9 release in the co-cultures and from CM-stimulated monocytic cells. Further experiments suggested that the stimulating factor present in CM was not TNF-α, but acted synergistically with autocrine monocyte-derived TNF-α to release monocytic proMMP-9. Thus, ovarian cancer cells can stimulate monocytic cells in vitro to make proMMP-9 without affecting the expression of its inhibitor TIMP-1. This induction is mediated via a soluble factor (provisionally named MMPSF) that requires synergistic action of autocrine or paracrine TNF-α.

**Keywords:** tumour necrosis factor alpha; MMP-9; monocytes; ovarian cancer

Matrix metalloproteases (MMPs) are a family of structurally and functionally related endopeptidases. They have in common a zinc ion at the active site and are released as an inactive pro-form (zymogen). Proteolytic activation enables MMPs to degrade components of the extracellular matrix, such as collagens, fibronectin and laminin (for review Matrisian, 1990, 1992; Woessner, 1991; Mauch et al, 1994; Murphy, 1995). Of the MMPs cloned so far, the gelatinases MMP-2 and MMP-9 degrade in vitro native type IV collagen, the main constituent of the basement membrane. MMP activity is controlled at several levels. Gene expression is regulated by cytokines, such as tumour necrosis factor alpha (TNF-α), transforming growth factor beta (TGF-β) and interferons (for review Matrisian, 1990, 1992; Woessner, 1991; Mauviel, 1993; Mauch et al, 1994; Murphy, 1995), activation of MMPs can be triggered in vitro by proteases and other MMPs (Matrisian, 1990, 1992; Woessner, 1991; Mauch et al, 1994; Murphy, 1995; Sang et al, 1995) and, finally, their proteolytic activity is counterbalanced by tissue inhibitors of metalloproteases (TIMPs) (Matrisian, 1990, 1992; Woessner, 1991; Mauch et al, 1994; Murphy, 1995).

MMPs play an important role in tissue remodelling during embryogenesis and wound healing (Matrisian, 1990, 1992; Bullen et al, 1995). In addition, these enzymes contribute to the pathology of chronic diseases, such as osteo- and rheumatoid arthritis (Woessner, 1991; Woessner and Gunja Smith, 1991; Stetler Stevenson, 1996), and malignancy (Liotta and Stetler Stevenson, 1991; Woessner, 1991; Stetler Stevenson, 1996). Events such as angiogenesis, intra- and extravasation and migration of tumour or host immune cells have been associated with MMP activity (Liotta and Stetler Stevenson, 1991; Karelina et al, 1995).

MMP-2 and -9 are present in biopsies of breast, bladder, ovarian, colorectal and prostate cancer and their levels seem to be related to tumour grade and invasion (Davies et al, 1993*a* and *b*; Hamdy et al, 1994; Naylor et al, 1994; Liabakk et al, 1996). However, relatively little is known about the mechanisms leading to MMP expression in vivo. A recently published report describes the interaction of T cells and monocytes leading to MMP-9 release (Kiener et al, 1995). A soluble factor, gp39, derived from T cells was found to be responsible for triggering MMP-9 production via monocytic CD40, the gp39-receptor.

The aim of our study was to investigate the interactions between human tumour cells and macrophages that lead to MMP-9 release. In previous work on biopsies of human ovarian cancer, the type IV gelatinases MMP-2 and MMP-9 were detected by zymography and their expression localized by in situ hybridization (Naylor et al, 1994). MMP-2 mRNA was found exclusively in the tumour stroma, whereas MMP-9 expression was discrete and seen in both tumour and stromal areas. Immunohistochemical studies using an antibody to the macrophage marker CD68 showed a positive correlation with the pattern found for MMP-9, which suggested that tumour-associated macrophages (TAMs) may be the source of MMP-9 (Naylor et al, 1994). TNF-α expression, as assessed by in situ hybridization, was confined to epithelial tumour areas, whereas immunoreactive TNF-α protein was found in both tumour and stromal areas. In this respect, the pattern of TNF-α protein was similar to that found for infiltrating macrophages (Naylor et al, 1993). These results suggested that TAMs could be the source of MMP-9 and that TNF-α might play a role in its production (Naylor et al, 1993, 1994).

In this report, we describe a mechanism that leads to monocytic proMMP-9 but not to TIMP-1 production in the presence of ovarian cancer cells. We provide evidence that a tumour-derived

*Received 5 January 1998*
*Revised 4 March 1998*
*Accepted 5 March 1998*

*Correspondence to:* TM Leber soluble factor, tentatively named MMPSF (matrix metalloproteinase-stimulating factor) demonstrates synergy with autocrine or paracrine TNF-α to stimulate MMP-9 release.

### MATERIAL AND METHODS

### Cell culture techniques

The human cell line PEO-1 was derived from ascites of a patient with a poorly differentiated adenocarcinoma before chemotherapy (Langdon et al, 1988). The cell line was maintained in RPMI (Gibco, Paisley, UK) supplemented with 10% fetal calf serum (FCS, Gibco) and 10 μg $ml^{-1}$ bovine insulin (Sigma, Poole, UK) and routinely passaged two to three times per week. The human monocytic cell line THP-1 was from ATCC (American Type Culture Collection, Rockville, IL, USA) and maintained at a cell concentration between 0.5 and $1\times10^6$ cells $ml^{-1}$ in RPMI containing 10% FCS and 50 μM beta-mercaptoethanol (Sigma). All cells were grown in Nunc tissue culture flasks and incubated in a humidified atmosphere at 37°C, 5% carbon dioxide.

### Experimental cell culture conditions and preparation of conditioned medium

PEO-1 cells were grown to near confluency, detached from the tissue culture flask with trypsin/versene (Gibco), resuspended in culture medium, pelleted (210 ***g***, 5 min), washed up to three times in phosphate-buffered saline (PBS) and resuspended in FCS-free Aim V medium (Gibco). Similarly, THP-1 cells were pelleted (210 ***g***, 5 min) and resuspended in FCS-free Aim V. Cells were counted using a haemocytometer and, if not otherwise stated, the cell concentration adjusted to $1\times10^6$ cells $ml^{-1}$. All experiments were set up in 24- or 96-well plates (Costar, Cambridge, MA, USA) and cell culture supernatant harvested, unless stated otherwise, after an incubation period of 48 h. Supernatant was cleared of cells and cell debris by centrifugation at 14 000 r.p.m. in a microfuge before storage at –20°C or immediate use for zymography. For use as conditioned medium (CM), the supernatant was sterile filtered (Acrodisc 0.2 μm, Gelman Sciences, Ann Arbor, MI, USA) before freeze-storage or use.

### Isolation of peripheral blood monocytes

Peripheral blood (50 ml) was taken from healthy volunteers by venupuncture, mixed with 5 ml of 3.8% sodium citrate and centrifuged (20 min, 300 ***g***) to obtain a cell pellet and platelet-rich plasma (PRP). PRP was centrifuged twice (2000 ***g***, 10 min) to remove the platelets (PPP, platelet-poor plasma) and stored on ice for later use. The cellular pellet was resuspended in 0.9% sodium chloride to 45 ml and erythrocytes precipitated with 5 ml of 6% Dextran T-500 (Pharmacia, Uppsala, Sweden). The lymphocyte-rich supernatant was transferred into a fresh tube, the cells pelleted by centrifugation (5 min, 200 ***g***), washed three times in wash buffer (0.9% sodium chloride, 10% PPP) and resuspended in 8 ml of PPP. Two millilitres of cell suspension were under layered with 2 ml of 42% Percoll (Pharmacia) prepared with PPP. After centrifugation (10 min, 300 ***g***) the monocyte-rich interphase was harvested by aspiration, washed three times in wash buffer and resuspended in Aim V medium. The cells were seeded and after an incubation period of 1 h at 37°C washed three times with Aim V medium to select the monocytes by adhesion. To assess the purity of the preparation, an aliquot of cells was set to adhere on a Petri dish, washed in the same way as the cells used for the stimulation experiments, air dried, stained using the α-naphthyl acetate esterase method (Yam et al, 1971) and counterstained with Meyers haematoxylin. The ratio of monocytes vs non-monocytes was determined by phase-contrast microscopy. In all experiments, purity of monocytes exceeded 90%.

### Endotoxin assessment

All solutions and buffers (RPMI, PBS, AIM V, water, CM, etc.) used in cell culture were checked for endotoxin content using a kinetic turbidity assay (BioWhittaker, Reading, UK) or the endotoxin detection kit (0.50 EU $ml^{-1}$) purchased from Associates of Cape Cod (Woods Hole, MA, USA). Levels were found to be below 100 pg $ml^{-1}$. Dose–response experiments of THP-1 cells to three types of endotoxin (*E. coli* 055:B5, 0111:B4 and *Salmonella minnesota*) showed that levels of 100 pg $ml^{-1}$ or less did not stimulate MMP-9 release in a detectable manner (data not shown). To achieve levels of MMP-9 similar to those obtained in co-culture or CM experiments with THP-1 (> fivefold background level), 1 ng $ml^{-1}$ or

**Figure 1** ProMMP-9 production was increased in co-cultures of the monocytic THP-1 cell line and the ovarian cancer cell line PEO-1 (1:1 ratio). THP-1 and PEO-1 cells were seeded separately or in co-culture at a 1:1 ratio. The cell culture supernatant was harvested after 48 h and the proMMP-9 production assessed by quantitative zymography. The left panel shows a typical zymogram of cell culture supernatants, the right panel a Western blot for MMP-9. **B** shows the quantitation of zymograms with respect to proMMP-9. In co-culture, proMMP-9 stimulation exceeded background levels five- to eightfold. Only proMMP-9 (92 kDa) can be detected. Left panel: lane 1, purified MMP-9; lane 2, THP-1 supernatant; lane 3, PEO-1 supernatant; lane 4, THP-1/PEO-1 co-culture supernatant. Right panel: lane 1, purified MMP-9; lane 2, THP-1 stimulated with CM derived from PEO-1 cells; lane 3, THP-1/PEO-1 co-culture supernatant. Statistical analysis of **B** using Students *t*-test: THP-1 vs THP-1/PEO-1, $P = 0.039$; PEO-1 vs THP-1/PEO-1, $P = 0.034$

© Cancer Research Campaign 1998

more of endotoxin was needed. Endotoxin levels present in FCS were assessed by Gibco and were found to be below 100 pg ml$^{-1}$.

### Zymogram analysis

Quantitative gelatinolytic zymography was performed according to an improved protocol described recently (Leber and Balkwill, 1997). For gel to gel comparison, a standard of commercially available purified human proMMP-9 (TCS Biologicals, Botolph Claydon, UK) was loaded on each gel in duplicate. All samples were assessed in the linear range of the assay and the individual MMP-9 activity expressed in ng (MMP-9) per µl (supernatant). All values of MMP-9 activity were based on at least two independent experiments. The error bars reflect the standard deviation.

### Spin column experiment

SpinColumns-30 (Clontech, Basingstoke, UK) were pre-spun twice for 3 min (4°C, 1100 *g*) to remove the equilibration buffer. A 50-µl aliquot of sample was loaded on the spin column and spun for 5 min (4°C, 1100 *g*). The flow-through was harvested and the volume adjusted to 55 µl with sterile PBS.

### Immunoprecipitation of TNF-α

The monoclonal anti-TNF-α antibody 6H11 (kindly provided by Dr N-B Liabakk, Trondheim, Norway) was added to CM or control samples at a concentration of 5 µg ml$^{-1}$. Samples were incubated for at least 1 h at 4°C on a rotor, then 40 µl of protein-G Sepharose (Sigma) added and samples incubated as before. To pellet the beads, samples were spun for 5 min at maximal speed in a microfuge and the supernatant carefully removed. To assess the MMP-9-inducing activity, the sample was added to THP-1 cells in a 1:10 dilution. To check for the efficiency and specificity of TNF-α precipitation, samples spiked with recombinant human TNF-α at a concentration of 10 ng ml$^{-1}$ were prepared. Precipitation of TNF-α and removal of the anti-TNF-α antibody was complete and specific. The experiments have been repeated with a commercially available monoclonal anti-TNF-α antibody (R&D Systems Europe, Abingdon, UK) and an unrelated antibody of the same antibody isotype.

### RNA preparation and Northern blotting

Total RNA was isolated using TriReagent (Molecular Research Center, Cincinnati, OH, USA) according to the manufacturers instructions, 10 µg heated to 65°C for 5 min, resolved on a 1% denaturing formaldehyde–agarose gel and blotted onto a Hybond N$^+$ membrane (Amersham, Slough, UK) by capillary transfer as described elsewhere (Balkwill, 1991). RNA was UV cross-linked to the nylon membrane using a Stratalinker (Stratagene, Cambridge, UK) and blots prehybridized and hybridized as described (Balkwill, 1991). cDNA probes for human TNF-α, MMP-9 and β-actin were radioactively labelled ($^{32}$P-dCTP, Amersham) by random priming using the Prime-It or the RmT-Prime-It labelling kit (Stratagene) according to the manufacturers instructions. After hybridization, blots were washed twice for 10 min at room temperature in 2 × standard saline citrate (SSC), 0.1% sodium dodecyl sulphate (SDS), then incubated twice for 15 min in 0.1% SSC, 0.1% SDS at 65°C and finally washed for 10 min in 2 × SSC at room temperature. For detection, blots were wrapped

**Figure 2** ProMMP-9 activity in co-culture supernatants depended on the ratio of PEO-1 to THP-1 cells. Cells were diluted in doubling dilutions starting from 1 × 10$^6$ cells ml$^{-1}$ and added to 1 × 10$^6$ cells ml$^{-1}$ of the other cell line. After 48 h, the cell culture supernatant was assessed by quantitative zymography. Maximal proMMP-9 release was obtained at a 1:1 of ratio tumour cells and monocytes. (The graph shows a typical experiment out of four experiments performed.)

in Saranwrap (Dow Chemicals) and exposed at –70°C to BioMax X-ray film (Kodak) for 4 to 56 h.

### Detection of TNF-α protein

TNF-α protein was detected using a WEHI-164 based bioassay (Espevik and Nissen Meyer, 1986) or a commercially available ELISA (R&D Systems Europe). A standard curve using recombinant human TNF-α was set up, and validation of the assay showed that accurate results could be obtained in the range between 20 and 500 pg ml$^{-1}$ TNF-α for the bioassay and 15–1000 pg ml$^{-1}$ for the ELISA.

### Western blotting

Supernatants of THP-1/PEO-1 co-cultures and purified human proMMP-9 were resolved on a 10% SDS–PAGE under reducing or non-reducing conditions and electroblotted onto a nitrocellulose membrane (Amersham) for 2 h at 45 V. After blocking of the membrane with PBS containing 10% non-fat dry milk, the blots were incubated with a monoclonal anti-human MMP-9 antibody diluted 1:1000 (Ab-3, Oncogene Science) or 1:5000 (CA-209, kindly provided by Dr Raphael Fridman, Wayne State University, DT, USA) in PBS, 0.01% Tween-20. Immunodetection was performed using the enhanced luminescence kit ECL (Amersham) or with SuperSignal Ultra (Pierce, Chester, UK) according to the manufacturers instructions.

### Other materials

Recombinant TNF-α, kindly provided by Knoll, Friedrichshafen, Germany, was prepared as a 15 µg ml$^{-1}$ stock in PBS, 0.3% bovine serum albumin (Sigma) and stored at –20°C. The synthetic MMP inhibitor BB-2116 (a gift from British Biotech Pharmaceuticals, Oxford, UK) was prepared in dimethyl sulphoxide (DMSO) as a 100 mM stock solution and diluted in PBS to, if not stated differently, a concentration of 30 µM.

© Cancer Research Campaign 1998

**Figure 3** PEO-1-derived conditioned medium (CM) induced proMMP-9 production by THP-1 cells in a dose-dependent manner. PEO-1-derived CM was added to THP-1 cells in the dilutions indicated. After 48 h, the cell culture supernatant was assessd by quantitative zymography. CM stimulated MMP-9 release from THP-1 cells in a dose-dependent manner. For all further stimulation experiments, a 1:10 dilution of CM was used

**Figure 4** CM induced increased MMP-9 production in isolated peripheral blood monocytes. Peripheral blood monocytes (PBM) were isolated as described in Material and methods, stimulated with 10 × concentrated CM and the cell culture supernatant analysed after an incubation period of 48 h. The zymogram (left panel) shows two close together migrating activities that were both enhanced in supernatant of CM-stimulated monocytes. Both proteins were identified as MMP-9 by Western blotting using the monoclonal anti-MMP-9 antibody CA-209 (right panel). In other experiments, only a single band of proMMP-9 was detectable. Left panel: lane 1, proMMP-9; lane 2, unstimulated PBMs; lane 3, CM stimulated PBMs. Right panel: lane 1, proMMP-9; lane 2, CM-stimulated PBMs)

### RESULTS

### Co-culture of ovarian cancer cells and monocytic cells induces proMMP-9 independent from cell–cell contact

The co-culture system consisted of the ovarian cancer cell line PEO-1 and the monocytic cell line THP-1. As shown in Figure 1A and B, supernatants generated when these cells were cultured alone contained low levels of the 92-kDa form of MMP-9 as measured by quantitative zymography. However, co-culture at a 1:1 ratio of tumour cells to monocytes led to strong production of proMMP-9 in supernatants. The identity of MMP-9 was confirmed by Western blotting (Figure 1A, right panel). Experiments showed that this 1:1 ratio was optimal (Figure 2). For a fixed number of PEO-1 cells, proMMP-9 release increased with an increasing number of THP-1 cells. For a fixed number of THP-1 cells, proMMP-9 release slightly increased with an increasing number of PEO-1 cells. Use of cell culture inserts (Millicell-CM, Milipore), separating the two cell lines but allowing exchange of soluble factors, led to similar strong induction of proMMP-9 as co-cultures without the cell culture inserts (data not shown).

**Figure 5** Inhibition of MMP-9 release by an anti-TNF-α antibody. THP-1/PEO-1 co-cultures (**A**) and CM-stimulated THP-1 cells (**B**) were treated with the monoclonal anti-TNF-α antibody 6H11 and the cell culture supernatant assessed by quantitative zymography. The antibody inhibits proMMP-9 release in both THP-1/PEO-1 co-cultures and from CM-stimulated THP-1 cells. Statistical analysis using paired Student's *t*-test: (**A**) THP-1/PEO-1 vs THP-1/PEO-1 in presence of 5 μg ml$^{-1}$ TNF-α antibody, $P = 0.028$; THP-1/PEO-1 vs THP-1/PEO-1 in presence of 10 μg ml$^{-1}$ TNF-α antibody, $P = 0.044$. (**B**) THP-1 vs THP-1+CM, $P < 0.001$; THP-1 vs THP-1+CM+ TNF-AB, $P = 0.003$)

### MMP-9 is produced by the monocytic cells in the co-cultures

These results suggested that cell–cell contact was not required and that the monocytic cells were the source of proMMP-9. To test this further, we prepared conditioned medium (CM) from both PEO-1 and THP-1 cells. CM from THP-1 cells did not enhance MMP-9 release from PEO-1 (data not shown), whereas PEO-1-derived CM induced proMMP-9 production by THP-1 (Figure 3). This induction was dose dependent and CM even at a 1:60 dilution CM had a clear proMMP-9-inducing effect on THP-1 cells. The identity of the protein induced by CM was confirmed as MMP-9 by Western blotting (Figure 1A, right panel). Cell counting experiments showed that the cell numbers in CM-stimulated and control cultures were not significantly different (data not shown).

### Peripheral blood monocytes (PBM) also produce proMMP-9 in response to CM

Similar to the monocytic cell line THP-1, isolated peripheral blood monocytes released increased amounts of proMMP-9 in response to CM. However, to achieve the level of induction shown (Figure 4), CM had to be concentrated 10-fold by ultrafiltration (NanoSpin Plus 10000 MWCO, Gelman Sciences, Ann Arbor, MI, USA).

### TNF-α is involved in tumour-stimulated monocytic MMP-9 production

Several lines of evidence suggested an involvement of the cytokine TNF-α in monocytic production of MMP-9. First, recombinant TNF-α induced proMMP-9 production by THP-1 cells in a dose-dependent

© *Cancer Research Campaign 1998*

**Figure 6** BB-2116 inhibits TNF-α release from LPS-stimulated THP-1 cells and proMMP-9 release from CM-stimulated THP-1 cells or in co-cultures of THP-1 and PEO-1. BB-2116 was added to LPS (100 ng $ml^{-1}$)-stimulated THP-1 cells (**A**), THP-1/PEO1 co-cultures (**B**) and CM-stimulated THP-1 cells (**C**). The cell culture supernatant was assayed for TNF-α (**A**) and MMP-9 (**B** and **C**) after an incubation period of 48 h. The matrix metalloproteinase inhibitor BB-2116 inhibits TNF-α release from LPS-stimulated THP-1 cells in a dose-dependent manner (**A**: ■, LPS + BB-2116; ▲, LPS; ●, unstimulated). The $IC_{50}$ was 300 nM as determined by computer-assisted curve fitting. Further, BB-2116 inhibits proMMP-9 release in a dose-dependent manner in both co-cultures (**B**: $IC_{50}$ 590 nM, ■, BB-2116; ●, control) and CM-stimulated THP-1 cells (**C**, $IC_{50}$ 860 nM, ■, CM + BB-2116; ●, CM; ▲, unstimulated)

manner. ProMMP-9 release was first detected at 0.3 ng $ml^{-1}$ TNF-α and reached its maximum at 10 ng $ml^{-1}$ TNF-α (data not shown). This finding is consistent with published results (Okada et al, 1990; Mauviel, 1993). TNF-α (10 ng $ml^{-1}$) had, however, a minimal effect on MMP-9 release from PEO-1 cells (data not shown). Second, a monoclonal antibody that neutralized TNF-α bioactivity inhibited MMP-9 release in co-cultures and also in CM-stimulated THP-1 cultures (Figure 5A and B).

In accordance with published results (Gearing et al, 1994, 1995), we found that the matrix metalloprotease inhibitor BB-2116 blocked shedding of TNF-α from its membrane-spanning precursor after LPS stimulation of THP-1 cells (Figure 6A, $IC_{50}$ 300 nM). This MMPI inhibited proMMP-9 production with a similar $IC_{50}$ in co-culture and in CM experiments (Figure 6B and C, $IC_{50}$ 590 nM and 860 nM). At the concentrations used in these experiments, BB-2116 had no cytotoxic or cytostatic effects on THP-1 cells as assessed by cell counting experiments (data not shown) and phase-contrast microscopy. Further, BB-2116 did not interfere with zymogram analysis of proMMP-9 in the assessed range (< 300 mM, data not shown). The observation that an inhibitor of TNF-α release also inhibited proMMP-9 release from monocytic cells when stimulated with CM led us to investigate a role for autocrine monocytic TNF-α production in proMMP-9 release.

### Analysis of MMP-9, TIMP-1 and TNF-α gene expression and protein

MMP-9 and TNF-α gene expression and protein production was studied in CM-stimulated and -unstimulated THP-1 cells over 48 h of incubation. Using Northern analysis, MMP-9 mRNA was detected in both CM-stimulated and -unstimulated THP-1 cells at 6 h. In unstimulated cells, the signal remained weak and decreased to below the detection limit after 24 h of incubation (Figure 7A). In CM-stimulated cells, however, MMP-9 expression was strongly induced, peaked at 14 h and remained strong until 48 h (Figure 7A). This finding is consistent with protein data obtained by zymography. First, proMMP-9 proteolytic activity was detected in the supernatant after 12 h, and levels seemed to increase steadily thereafter (Figure 7B).

Reprobing the above blot for TNF-α mRNA showed gene expression in both CM-stimulated and -unstimulated THP-1 cells with the same time course and level of expression (Figure 7A). TNF-α mRNA was not detectable at the beginning of culture, peaked at 2 h and declined thereafter. TNF-α protein, as detected by bioassay or ELISA in three independent experiments, followed the pattern observed for mRNA. Maximal TNF-α protein was detected 5–8 h after stimulation (60 pg $ml^{-1}$ and 21 pg $ml^{-1}$ as determined by bioassay and ELISA respectively). No TNF-α was detected at the beginning of the incubation period and the amounts decreased to the detection limit (ELISA, 15 pg $ml^{-1}$) or below (bioassay, 19 pg $ml^{-1}$; data not shown) by 16 h.

Reprobing of the same blot for TIMP-1 mRNA showed no difference in TIMP-1 expression between unstimulated and CM-stimulated THP-1 cells (Figure 7A). Reprobing of the blot with β-actin showed that loading was even (Figure 7A).

Thus, these experiments showed that THP-1 cells release low amounts of TNF-α even without stimulation with CM.

### The proMMP-9-stimulating activity (MMPSF) present in CM is distinct from TNF-α

The level of TNF-α in different batches of CM was measured by bioassay. This varied from 0 to 160 pg $ml^{-1}$ without detectable

© Cancer Research Campaign 1998

**Figure 7** Kinetics of proMMP-9, TNF-α and TIMP-1 gene expression and protein production in unstimulated and CM-stimulated THP-1 cells. THP-1 cells were stimulated 1:10 with PEO-1-derived conditioned medium (CM) or remained unstimulated. MMP-9, TIMP-1, β-actin and TNF-α gene expression was analysed by Northern blotting (**A**). MMP-9 expression was strong and sustained in CM-stimulated THP-1 cells only. TNF-α and TIMP-1 gene expression showed a similar pattern in both CM-stimulated and -unstimulated THP-1 cells. Probing for β-actin was performed as for loading control. MMP-9 protein was analysed by zymography (**B**: ■, unstimulated THP-1 control; ●, CM-stimulated THP-1 cells), TNF-α protein by bioassay or ELISA (data not shown)

changes in the ability of CM to induce monocytic proMMP-9 (data not shown). Further, immunoprecipitation of potential TNF-α from CM had only a minor effect on its MMP-9-stimulating activity (Figure 8A). Controls showed that recombinant TNF-α could be precipitated efficiently and the TNF-α antibody 6H11 was fully removed by protein-G sepharose precipitation. In addition, preparation of CM in the presence of 30 µM BB-2116, which blocks TNF-α shedding from the cell surface (Figure 5A), followed by removal of BB-2116 by gel filtration did not affect MMP-9 release when this CM was added to the monocytic cells (Figure 8B). Taken together, these results suggested that tumour cell-derived TNF-α did not play a major role in stimulation of monocytic MMP-9 production.

### The tumour-derived soluble factor (MMPSF)

Our conclusion from the above experiments was that a tumour cell-derived soluble factor was synergistic with endogenous monocytic-produced TNF-α to stimulate proMMP-9 production. The factor was heat sensitive; heating to 85°C for 30 min completely abolished its ability to induce MMP-9 (data not shown). Preliminary gel filtration experiments indicated a peak of activity with an apparent molecular mass of 89 kDa. This result was further evidence that MMPSF was distinct from TNF-α, as the molecular mass of TNF-α has been reported to be 45 kDa by gel filtration (Aggarwal et al, 1984). A model summarizing our results is shown in Figure 9.

© Cancer Research Campaign 1998

**Figure 8** The MMP-9 release stimulating activity in CM is not TNF-α. Immunoprecipitation of TNF-α potentially present in PEO-1-derived CM only slightly altered its capacity to induce MMP-9 release from THP-1 cells (**A**). Controls showed that recombinant TNF-α could be precipitated efficiently and the TNF-α antibody 6H11 was fully removed by protein-G sepharose precipitation. Control, unstimulated THP-1 cells; AB, TNF-α antibody 6H11; IP, immunoprecipitation; AimV, culture medium; prot-G Seph, protein-G Sepharose; C-AB, control antibody of same IgG-isotype as 6H11 ($IgG_1$). Statistical analysis using Student's *t*-test: CM vs CM+AB, $P < 0.001$; CM+AB+IP vs CM+AB, $P = 0.002$). (**B**) Conditioned medium from PEO-1 cells was prepared in the presence of BB-2116 (30 μM). Addition of this CM to THP-1 cells in a 1:10 dilution results in a 50% reduction of MMP-9 release from THP-1 cells. When BB-2116 was removed from CM by gel filtration, the inhibitory effect of BB-2116 disappeared and full MMP-9 stimulation was achieved. Statistical analysis using Student's *t*-test: control vs CM, $P = 0.009$; no spin column, CM vs CM+BB-2116 (3 μM), $P = 0.022$; after spin column, CM vs CM+BB-2116 (3 μM), $P$=0.251)

### DISCUSSION

In this study, we investigated the interactions between human ovarian cancer cells and monocytic cells with respect to MMP-9 production and the role of TNF-α in this process. Our results showed that co-cultures of the ovarian cancer cell line PEO-1 and the monocytic cell line THP-1 led to an increase of proMMP-9 in the cell culture supernatant. Further experiments indicated that the monocytic cells were the source of proMMP-9 and that a soluble factor, MMPSF, present in tumour-derived conditioned medium (CM) was sufficient to induce proMMP-9 production in both the monocytic cell line THP-1 and peripheral blood monocytes. Experiments with neutralizing antibodies to TNF-α, the inhibitor BB-2116, which blocks shedding of TNF-α from its membrane spanning precursor, and Northern analysis revealed that autocrine TNF-α production by the monocytic cells was necessary for the synthesis of monocytic proMMP-9. These experiments also showed that, in contrast to MMP-9 production, TIMP-1 expression remained unchanged. Finally, MMPSF was found to be distinct from TNF-α and that the synergistic action of both MMPSF and TNF-α was required for monocytic proMMP-9 production.

This study was based on observations made on biopsies of human ovarian cancer. In situ hybridization and immunohistochemical studies have revealed similarities in the expression pattern of MMP-9 mRNA and the distribution of tumour-associated macrophages (TAMs) (Naylor et al, 1994). Further, TNF-α expression has been localized by in situ hybridization to tumour areas (Naylor et al, 1994), whereas immunohistochemical evidence has suggested localization of TNF-α protein and TAMs (Naylor et al, 1993). To investigate the relationship between monocytes, tumour cells, TNF-α and MMP-9, we established a co-culture system consisting of the human ovarian cancer cell line PEO-1 and the human monocytic cell line THP-1 or isolated peripheral blood monocytes. We observed that MMP-9 was released by the monocytic cells and found that a soluble, tumour cell-derived factor, MMPSF, was responsible for this MMP-9 production. These findings are consistent with the in vivo observations (Naylor et al, 1993, 1994). Detection of TNF-α mRNA and protein in the in vitro system showed that the monocytic cell line THP-1 produced TNF-α. In vivo observations, however, have localized TNF-α gene expression to the tumour area (Naylor et al,

© Cancer Research Campaign 1998

**Figure 9** Model of stimulation of monocytic MMP-9 release. A soluble protein derived from ovarian cancer cells acts synergistically with autocrine/paracrine TNF-α, leading to monocytic proMMP-9 production

1994) but TNF-α protein to TAMs. Therefore, in vivo, cells other than the monocytes might be the source of TNF-α protein. This interpretation would also explain the need to concentrate CM to achieve proMMP-9 production by peripheral blood monocytes. In summary, we suggest that, in ovarian cancer, TNF-α gene expression in epithelial tumour areas leads to low levels of tissue TNF-α, which promotes, together with tumour-derived MMPSF, monocytic proMMP-9 production. Further experiments have confirmed this hypothesis. THP-1 cells were preincubated for 16 h. At this timepoint, THP-1 cells ceased to produce TNF-α, and there was no detectable TNF-α in the culture medium. Under these circumstances, CM did not induce proMMP-9. In addition, CM-induced MMP-9 production does not affect TIMP-1 gene expression, suggesting a net production of proteolytic activity. Further work is required on the observation that, exclusively, the pro-form of MMP-9 (92 kDa) was detected in the cell culture supernatants.

Production of monocytic proMMP-9 has been the focus of several recent papers. T-cell-derived gp-39 (Kiener et al, 1995), TNF-α and IL-1β (Sarén et al, 1996) were shown to be potent inducers of monocytic proMMP-9 production. The identity of MMPSF present in CM from tumour cells remains to date unknown. TNF-α was a potential candidate but the experiments performed showed that MMPSF is distinct from TNF-α. So far, we have found that MMPSF is heat sensitive, and the observation that BB-2116 did not inhibit its release eliminates a series of proteins for which such an effect has been reported (e.g. TGF-α, M-CSF, FasL, etc. (Crowe et al, 1995; Kayagaki et al, 1995; Mullberg et al, 1995; Bennett et al, 1996; Couet et al, 1996; Drummond and Gearing, 1996; Feehan et al, 1996). Another parameter was obtained by gel filtration of CM revealing a peak of activity with a molecular mass of 89 kDa. This excludes IL-1β, another potent stimulus of monocytic proMMP-9 production, as IL-1β shows a molecular mass of 17.5 kDa by gel filtration (Schmidt, 1984; Gery and Schmidt, 1985). The nature of MMPSF and its mechanism of action are the main focus of our current work.

Our results further suggest a new mechanism of action of synthetic MMPIs in cancer. MMPIs were originally designed to reduce excessive MMP activity due to an imbalance of MMPs and their natural inhibitors (TIMPs). However, synthetic MMPIs also inhibit shedding of TNF-α from its membrane spanning precursor (Gearing et al, 1994, 1995) and of other cytokines: TGF-α, FasL, IL-6 receptor, stem cell factor, M-CSF, TNF-α receptors, L-selectin and thyrotropin receptor ectodomain (Crowe et al, 1995; Kayagaki et al, 1995; Mullberg et al, 1995; Bennett et al, 1996; Couet et al, 1996; Drummond and Gearing, 1996; Feehan et al, 1996). Therefore, inhibition of release of cytokines that play a role in tumour development represents an alternative or additional mechanism of action of MMPIs. This option might be of particular importance if the cytokine has stimulatory activity on MMP gene expression, as is the case for TNF-α. However, the $IC_{50}$ values of synthetic MMPIs for MMPs are generally 10- to 100-fold lower than those that influence cytokine release (Chirivi et al, 1994; Gearing et al, 1994). Hence, the effect might only be a secondary one to MMP inhibition. However, because of the broad activities of cytokines and their potency, even small changes might contribute to the observations made in animal models of human cancer (Talbot and Brown, 1996) or in current clinical trials.

In this study, we established a simple in vitro system that allowed us to analyse the mechanisms leading to monocytic MMP-9 production. Our in vitro findings were in keeping with the observations made on biopsy material of human ovarian cancer. Further studies are required to fully characterize MMPSF and to determine the biological role of the 92-kDa pro-form of MMP-9.

### ACKNOWLEDGEMENTS

We thank Dr R Fridman (Wayne State University, DT, USA) for kindly supplying the antibody to MMP-9, Dr G Wells and Dr L Bone (British Biotech Pharmaceuticals, Oxford, UK) for the MMP-9 cDNA and BB-2116, Knoll (Friedrichshafen, Germany) for the recombinant TNF-α and Dr N-B Liabakk (Trondheim, Norway) for the TNF-α antibody and her useful comments during the project. We would further like to acknowledge the technical assistance of Parames Thavasu and Chris Selkirk for assessment of TNF-α protein and LPS respectively. This project was supported by a grant from the European Union (Grant No: BIO4-CT96-5050).

### REFERENCES

Aggarwal BB, Moffat B and Harkins RN (1984) Human lymphotoxin. Production by a lymphoblastoid cell line, purification, and initial characterization. *J Biol Chem* **259**: 686–691

© Cancer Research Campaign 1998

Balkwill F (1991) *Cytokines, A Practical Approach*. The Practical Approach Series. Oxford University Press: Oxford

Bennett TA, Lynam EB, Sklar LA and Rogelj S (1996) Hydroxamate-based metalloprotease inhibitor blocks shedding of L-selectin adhesion molecule from leukocytes: functional consequences for neutrophil aggregation. *J Immunol* **156**: 3093–3097

Bullen EC, Longaker MT, Updike DL, Benton R, Ladin D, Hou Z and Howard EW (1995) Tissue inhibitor of metalloproteinases-1 is decreased and activated gelatinases are increased in chronic wounds. *J Invest Dermatol* **104**: 236–240

Chirivi RG, Garofalo A, Crimmin MJ, Bawden LJ, Stoppacciaro A, Brown PD and Giavazzi R (1994) Inhibition of the metastatic spread and growth of B16-BL6 murine melanoma by a synthetic matrix metalloproteinase inhibitor. *Int J Cancer* **58**: 460–464

Couet J, Sar S, Jolivet A, Hai MT, Milgrom E and Misrahi M (1996) Shedding of human thyrotropin receptor ectodomain. Involvement of a matrix metalloprotease. *J Biol Chem* **271**: 4545–4552

Crowe PD, Walter BN, Mohler KM, Otten-Evans C, Black RA and Ware CF (1995) A metalloprotease inhibitor blocks shedding of the 80-kD TNF receptor and TNF processing in T lymphocytes. *J Exp Med* **181**: 1205–1210

Davies B, Brown PD, East N, Crimmin MJ and Balkwill FR (1993*a*) A synthetic matrix metalloproteinase inhibitor decreases tumor burden and prolongs survival of mice bearing human ovarian carcinoma xenografts (published erratum appears in *Cancer Res*, 1993, **53**: 3652). *Cancer Res* **53**: 2087–2091

Davies B, Waxman J, Wasan H, Abel P, Williams G, Krausz T, Neal D, Thomas D, Hanby A and Balkwill F (1993*b*) Levels of matrix metalloproteases in bladder cancer correlate with tumor grade and invasion. *Cancer Res* **53**: 5365–5369

Drummond AH and Gearing AJ (1996) Matrix metalloproteinases and disease (abstract). *FASEB J* **10**: 754

Espevik T and Nissen Meyer J (1986) A highly sensitive cell line. WEHI 164 clone 13, for measuring cytotoxic factor/tumor necrosis factor from human monocytes. *J Immunol Methods* **95**: 99–105

Feehan C, Darlak K, Kahn J, Walcheck B, Spatola AF and Kishimoto TK (1996) Shedding of the lymphocyte L-selectin adhesion molecule is inhibited by a hydroxamic acid-based protease inhibitor. Identification with an L-selectin-alkaline phosphatase reporter. *J Biol Chem* **271**: 7019–7024

Gearing AJ, Beckett P, Christodoulou M, Churchill M, Clements J, Davidson AH, Drummond AH, Galloway WA, Gilbert R, Gordon JL, Leber TM, Mangan M, Miller K, Nayee P, Owen K, Patel S, Thomas W, Wells G, Wood LM and Wooley K (1994) Processing of tumour necrosis factor-alpha precursor by metalloproteinases. *Nature* **370**: 555–557

Gearing AJ, Beckett P, Christodoulou M, Churchill M, Clements JM, Crimmin M, Davidson AH, Drummond AH, Galloway WA, Gilbert R, Gordon JL, Leber TM, Mangan M, Miller K, Nayee P, Owen K, Patel S, Thomas W, Wells G, Wood LM and Wooley K (1995) Matrix metalloproteinases and processing of pro-TNF-alpha. *J Leukoc Biol* **57**: 774–777

Gery I and Schmidt JA (1985) Human interleukin 1. *Methods Enzymol* **116**: 456–467

Hamdy FC, Fadlon EJ, Cottam D, Lawry J, Thurrell W, Silcocks PB, Anderson JB, Williams JL and Rees RC (1994) Matrix metalloproteinase 9 expression in primary human prostatic adenocarcinoma and benign prostatic hyperplasia. *Br J Cancer* **69**: 177–182

Karelina TV, Goldberg GI and Eisen AZ (1995) Matrix metalloproteinases in blood vessel development in human fetal skin and in cutaneous tumors. *J Invest Dermatol* **105**: 411–417

Kayagaki N, Kawasaki A, Ebata T, Ohmoto H, Ikeda S, Inoue S, Yoshino K, Okumura K and Yagita H (1995) Metalloproteinase-mediated release of human Fas ligand. *J Exp Med* **182**: 1777–1783

Kiener PA, Moran Davis P, Rankin BM, Wahl AF, Aruffo A and Hollenbaugh D (1995) Stimulation of CD40 with purified soluble gp39 induces proinflammatory responses in human monocytes. *J Immunol* **155**: 4917–4925

Langdon SP, Lawrie SS, Hay FG, Hawkes MM, McDonald A, Hayward IP, Schol DJ, Hilgers J, Leonard RC and Smyth JF (1988) Characterization and properties of nine human ovarian adenocarcinoma cell lines. *Cancer Res* **48**: 6166–6172

Leber TM and Balkwill FR (1997) Zymography: a single step staining method for quantitation of proteolytic activity on substrate gels. *Anal Biochem* **249**: 24–28

Liabakk NB, Talbot I, Smith RA, Wilkinson K and Balkwill F (1996) Matrix metalloprotease 2 (MMP-2) and matrix metalloprotease 9 (MMP-9) type IV collagenases in colorectal cancer. *Cancer Res* **56**: 190–196

Liotta LA and Stetler Stevenson WG (1991) Tumor invasion and metastasis: an imbalance of positive and negative regulation. *Cancer Res* **51**: 5054s–5059s

Matrisian LM (1990) Metalloproteinases and their inhibitors in matrix remodeling. *Trends Genet* **6**: 121–125

Matrisian LM (1992) The matrix-degrading metalloproteinases. *Bioessays* **14**: 455–463

Mauch C, Krieg T and Bauer EA (1994) Role of the extracellular matrix in the degradation of connective tissue. *Arch Dermatol Res* **287**: 107–114

Mauviel A (1993) Cytokine regulation of metalloproteinase gene expression. *J Cell Biochem* **53**: 288–295

Mullberg J, Durie FH, Otten Evans C, Alderson MR, Rose John S, Cosman D, Black RA and Mohler KM (1995) A metalloprotease inhibitor blocks shedding of the IL-6 receptor and the p60 TNF receptor. *J Immunol* **155**: 5198–5205

Murphy G (1995) Matrix metalloproteinases and their inhibitors. *Acta Orthop Scand Suppl* **266**: 55–60

Naylor MS, Stamp GW, Foulkes WD, Eccles D and Balkwill FR (1993) Tumor necrosis factor and its receptors in human ovarian cancer. Potential role in disease progression. *J Clin Invest* **91**: 2194–2206

Naylor MS, Stamp GW, Davies BD and Balkwill FR (1994) Expression and activity of MMPS and their regulators in ovarian cancer. *Int J Cancer* **58**: 50–56

Okada Y, Tsuchiya H, Shimizu H, Tomita K, Nakanishi I, Sato H, Seiki M, Yamashita K and Hayakawa T (1990) Induction and stimulation of 92-kDa gelatinase/type IV collagenase production in osteosarcoma and fibrosarcoma cell lines by tumor necrosis factor alpha. *Biochem Biophys Res Commun* **171**: 610–617

Sang QX, Birkedal Hansen H and Van Wart HE (1995) Proteolytic and non-proteolytic activation of human neutrophil progelatinase B. *Biochim Biophys Acta* **1251**: 99–108

Sarén P, Welgus HG and Kovanen PT (1996) TNF-alpha and IL-1beta selectively induce expression of 92-kDa gelatinase by human macrophages. *J Immunol* **157**: 4159–4165

Schmidt JA (1984) Purification and partial biochemical characterization of normal human interleukin 1. *J Exp Med* **160**: 772–787

Stetler Stevenson WG (1996) Dynamics of matrix turnover during pathological remodeling of the extracellular-matrix. *Am J Pathol* **148**: 1345–1350

Talbot DC and Brown PD (1996) Experimental and clinical studies on the use of matrix metalloproteinase inhibitors for the treatment of cancer. *Eur J Cancer* **32A**: 2528–2533

Woessner JF Jr (1991) Matrix metalloproteinases and their inhibitors in connective tissue remodeling. *Faseb J* **5**: 2145–2154

Woessner JF Jr and Gunja Smith Z (1991) Role of metalloproteinases in human osteoarthritis. *J Rheumatol Suppl* **27**: 99–101

Yam LT, Li CY and Crosby WH (1971) Cytochemical identification of monocytes and granulocytes. *Am J Clin Pathol* **55**: 283–290

© Cancer Research Campaign 1998